# Nanomedicine: a primer for surgeons

**DOI:** 10.1007/s00383-012-3162-y

**Published:** 2012-08-15

**Authors:** K. K. Y. Wong, X. L. Liu

**Affiliations:** Department of Surgery, LKS Faculty of Medicine, The University of Hong Kong, Queen Mary Hospital, Pokfulam Road, Hong Kong SAR, China

**Keywords:** Nanoparticles, Nanodiagnostics, Nanomedicine, Therapeutics

## Abstract

The advances in science have resulted in the emergence of nanotechnology, which deals with the design and use of tools and devices of size 1–100 nm. The application of nanotechnologies to medicine is thus termed nanomedicine. Significant research has been focused on this new and exciting field and this review article will describe the basics of nanomedicine. This is followed by its experimental and clinical applications in diagnostics, drug therapy and regenerative medicine. Safety issues of in vivo use of nanomaterials are also discussed. In the future, it is foreseen that nanomedicine will facilitate the development of personalized medicine and will have a major impact on the delivery of better healthcare.

## Introduction

Nanotechnology is defined as the design, characterization and application of structures, devices and systems by controlling shape and size at nanometer scale level (ranging from 1 to 100 nm) [1]. A nanometer is one billionth of a meter (10^−9^ m). Putting this into biological context, the width of DNA is approximately 2.5 nm and protein molecules measure 1–20 nm. This new technology has already been widely used in microelectronics, materials manufacture, robotics, and dye processing.

**Fig. 1:**
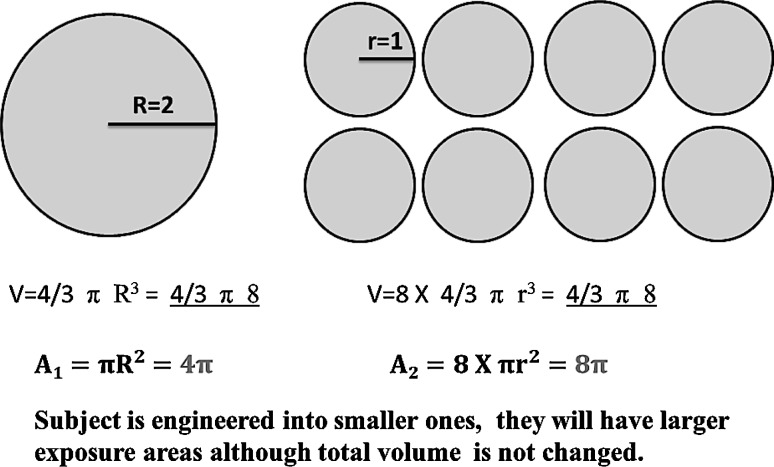
Schematic diagram to demonstrate the effect of smaller particle size on the surface area to volume ratio. Once subject was manufactured into smaller ones, the total surface area will be significant larger than original one. Therefore, the ratio of surface to volume will also be increased

When larger micro/macro conventional materials are engineered into nanosized particles and materials, completely different physiochemical and biological properties are seen (Fig. 1) [2, 3]. Furthermore, as many molecules involved in biological events interact fundamentally at the “nano” level, nanomaterials engineered are thus believed to be able to modulate or change the biological processes at the cellular level [4, 5].

In recent years, nanotechnology has extended into the field of medicine and this new discipline has been termed “Nanomedicine”. In contrast to conventional therapies where the basic approach is to remove diseased cells faster than healthy cells, nanomedicine attempts to use sophisticated approaches to either kill specific cells or repair them, one cell at a time. With rapid development of nanomedicine, sub-branches in nanooncology [6–8], nanoneurology [9–12], nanocardiology [13–15], nanoorthopedics [16–19], as well as nanoophthalmology [20] have also emerged. In terms of clinical applications, nanomedicine is providing a new and promising prospect for diagnosis and therapy. For instance, some nanomaterials have the potential to modulate and reduce the immune response to foreign tissues such as breast implants, which will contribute better outcomes like faster healing, better cosmetics, as well as less foreign body response [21]. Furthermore, nanotechnology has also been incorporated in the field of tissue engineering and reconstruction, which will greatly enhance and contribute to the field of regenerative medicine [22]. Nanomedicine thus offers new possibilities towards the development of personalized medicine, so as to allow improved treatment efficacies for many diseases.

In this review, we will describe relevant research and clinical application of nanotechnology and nanomaterials in clinical diagnosis and therapy.

## Nanomedicine in diagnosis

Early diagnosis plays an important role for the successful prevention and efficient treatment of diseases. This is particularly true in the case of cancer, as earlier diagnosis correlates with a significant increase in the cure rate. Furthermore, more information about the molecular mechanisms of the pathophysiology will lead to the development of newer and better anticancer drugs. The advantage of nanoparticle-based diagnostics lies in their higher sensitivity and selectivity when compared to classical methods.

### MRI contrast

Magnetic resonance imaging (MRI) is an important diagnostic imaging tool in the management of cancer. The addition of contrast agents like gadolinium in MRI helps to enhance image quality. Nanoparticles present a new collection of contrast agents. For example, gadolinium-based dendrimers is an enhancement of the traditional gadolinium, and can be effective at a very low concentration. A number of different dendrimers can target different organs. Furthermore, iron oxide nanoparticles can generate superior signal and have been used in liver imaging and for cell tracking studies. As they are metabolized through endogenous iron salvage pathways, they have already been introduced as clinical contrast agents.

Some nanodelivery devices implanted in body, not only contributes to carry and release of therapeutic drugs molecules, but also can be used for in vivo imaging. Meanwhile, the self-assembling characteristics of nanodelivery devices in body, on one hand, could serve as a delivery system for improvement of therapeutic efficacy [23]. On the other hand, their location in body can be tracked and imaged easily and clearly by MRI due to metallic nature in some of nanodelivery devices. Furthermore, these containers can be inserted directly at the site of an injury or tumor tissue, which would act as biosensors within the body to clearly demonstrate illness change via signaling frequency, while this differential signaling frequency will be easily detected by MRI contrast. John et al. [24, 25] reported the application of magnetic nanoparticles with magnetomotive optical coherence tomography for imaging of mammary tumors in rats. They claimed dynamic magnetomotive imaging is capable of detecting very low concentrations of nanoparticles with low-intensity magnetic fields, which would present a clinical possibility to use MRI to identify tumor location followed by magnetomotive optical coherence tomography-guided biopsy or surgery [26]. So far some nanomaterials with better biocompatibility have been also used for imaging diagnosis, including iron oxide, manganese oxide, and composite nanomaterial. These nanometal materials are widely studied as carriers for delivery of anti-cancer drug; meanwhile, they are revealed as the important candidates in nanodiagnostic imaging materials, as they are biocompatible and have superior contrast effects in MRI assay [27–29].

### Fluorescent nanoparticles for surgery

In the past decades, resection of the tumor has been guided by pre-operative imaging and also surgeons’ ability to differentiate tumor from normal tissues during operative procedures. Ensuring an adequate margin during tumor excision and minimizing the destruction of excessive normal tissue is thus a great challenge. This is particularly true in brain tumor surgery, as it is widely known that accurate tumor delineation will help improve survival rate and quality of life after surgery. Staining of tumor tissue using fluorophores or visible dyes, such as fluorescein, indocyanine green, bromophenol blue, and Coomassie Blue [30–33] has been attempted in brain tumor surgery. Many limitations still exist, including requirement to special lighting and fast diminishing of fluorophores/dyes (short-lasting retention), as well as poor visual contrast or target specificity.

The new physiochemical and biomedical characteristics of nanomaterials have made it possible for fluorescent nanoparticles to solve these limitations or obstacles. Fluorescent nanoparticles have several advantages, including selective tumor targeting, tumor-specific targeting moiety, high loading and quality of contrast agents, non-toxic [34, 35]. In this regard, Veiseh et al. [36] conjugated iron oxide-based nanoparticles with near-infrared fluorescent dye Cy5.5 to form fluorescent nanoparticles as a new dye, and proposed that they could inhibit brain tumor cell proliferation and invasion36, afterwards, they further revealed this fluorescent nanoparticles could pass through blood–brain barrier and reach tumor site, as well as aggregation in local tumor tissue [37]. Furthermore, in order to enhance biocompatibility, stability in physiological solutions, nontoxicity, and the ability to traverse biological barriers, they again reported a polyethylene glycol (PEG)-mediated synthesis process, aiming to produce well-dispersed, ultrafine, and highly stable iron oxide nanoparticles for in vivo applications [38]. Despite deep tissue penetration capability of these fluorescent nanoparticles, they were only visualized under separate monitor and invisible to the naked eye. Orringer et al. targeted glioma to explore visual tumor delineation by using nanoparticles surface-conjugated F3 peptide, and they developed a polyacrylamide (PAA) nanoparticle containing blue dye, Coomassie Brilliant Blue G-250 (CB). There was a better visible color contrast enhancer for intraoperative tumor delineation, and was also safe for intravenous injection, even at high doses [39]. Recently Nie et al. engaged in in vivo experiment using these fluorescent nanoparticles and further revealed this tumor-targeting deep-blue nanoparticle-based visible contrast agent was effective in tumor-specific color staining [40].

### Nanoprobes/chips array technology

Nano-scale materials are the ideal candidates to be used as bio-probes in vitro as they are more sensitive to very small targets. Their enlarged volume/surface ratio, surface tailor-ability, multi-functionality and intrinsic optical properties offer remarkable opportunities to detect and monitor slight signal variation in complex biological environments [41, 42]. For instance, the larger surface area of nanomaterials greatly enhances attachment of target-specific molecules, which contributes to ultrasensitive detection [43]. Thus, accurate diagnosis becomes possible at the molecular or single cell level due to fast responses and improved sensitivity. The ultimate goal of in vitro diagnostics is a relatively non-invasive, early, and accurate detection of biological disease markers during screening. One clinical scenario is the accurate and rapid diagnosis of incubating respiratory viruses in pre-operative patients in an attempt to reduce post-operative complications. Currently, many nanoprobe platforms have been described.

One example is quantum dots (QDs). These are well-established nano-scale crystals composed of an inorganic elemental core like cadmium or mercury, and a surrounding metal shell with resistance to photo-bleaching, spread absorption spectra covering UV to near-infrared region, and long fluorescence lifetimes, as well as size-dependent optical properties. They have broad biomedical applications in cellular imaging, immunoassays, biosensors, and microfluidic protein chip detection [44–48]. Furthermore, the photo-physical properties of QDs enable them to effectively link with acceptors and amplify target signal via enhanced energy-transfer efficiency. These features allow QD-based nanoprobes to generate a very distinct signal, even in the case of low target abundance [49]. In the biomedical area, QD optical probes have been used in signal transduction due to their better fluorescence resonance energy transfer properties, and designed to detect the proteins and peptides, as well as those small molecules [50, 51].

## Nanomedicine in therapeutics

One advantage of nanoparticles for biomedical applications is their ability to overcome various biological barriers and to localize into the target tissue. The nanoparticles could be a vector/carrier or be the active drug itself. The targeting of specific tissues could be due to passive targeting or active localization using specific additional molecules, which allows for molecular recognition of the target tissue or for triggered release of the payload at the disease site. Furthermore, nano-formulations can provide sustained release profiles for up to 24 h, which can improve patient compliance.

### Nanocarriers for cancer drug delivery

Although chemotherapy has been one of the principal treatment modalities for cancer, efficacies are mostly unsatisfactory due to non-specific action, which results in significant systemic side effects [52–54]. Ideally, anti-cancer drug molecules should act specifically on target cancer cells and accumulate preferentially at target tissues in sufficient concentration [55, 56]. Therefore, improvement of delivery efficacy of anti-cancer drugs can enhance the selective cytotoxicity to tumor cells, and is essential to reducing side effects in the body.

Cancer vasculatures have unique characteristics in both morphological structure and physiological features. These include: (1) highly chaotic and irregular arrangement of blood vessels in contrast to normal ones [57, 58]; (2) cancer blood vessels has overabundance of anionic phospholipids and proteoglycans; (3) vascular networks in cancer tissue have increased permeability to circulating macromolecules compared with normal ones. The size of vascular gap openings to cancer tissue usually increases up to 400–600 nm [59], which is remarkably larger than blood vessels in normal tissues. Macromolecules and nanoparticle drug carriers engineered to a specific size can thus preferentially extravasate from leaky cancer vasculatures and accumulate in cancer tissue [60–62]—enhanced permeability and retention (EPR) effect. The EPR effect allows accumulation of anti-cancer drugs in cancer tissues, thereby allowing for effective anticancer therapy with minimal drug toxicity.

**Fig. 2:**
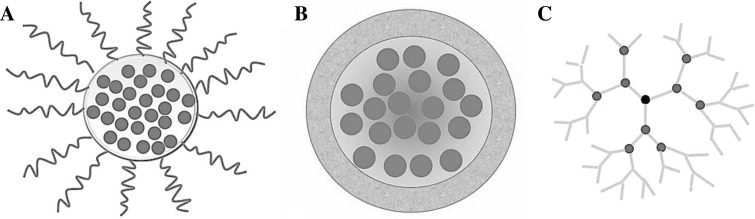
Schematic diagram showing various nano-delivery systems. **a** Polymicelles consist of a hydrophilic shell, which improves the biocompatibility and stability of the carrier in circulation, and a hydrophobic core for conjugation with hydrophobic anti-cancer drug molecules. **b** Liposomes have bimolecular lipid layers. The outer hydrophilic layer allows easy passage through cell membranes. The inner layer is hydrophobic and contains drug molecules. **c** Dendrimers are repetitively branched molecules typically symmetric around the core. Their “tree branch” like structure allows efficient drug carrying capacity and the ease of attaching functional groups for targeted delivery

So far a variety of nanoscale drug delivery systems have been developed. These nanosystems include polymeric micelles, liposomes, dendrimers, and nanoemulsions [63] (Fig. 2). Polymeric micelles are spherical and nanoscale colloidal carriers formed by the self-assembly of amphiphilic copolymers with both hydrophilic and hydrophobic segments in aqueous solution. These polymeric micelles are effective drug carriers for cancer therapy as they can incorporate water insoluble anti-cancer drugs in their hydrophobic core. Kataoka et al. found that the hydrophilic shell layer of these nano-carriers can prevent the incorporated drugs from degradation caused by enzymes and avoid non-selective uptake by macrophages distributed in whole body [64].

Liposomes are spherical self-closed structures consisted of concentric lipid bilayers. They have an inner aqueous compartment enclosed by those lipid bilayers which are similar to biological membranes. As a result of this, the solubility of hydrophobic chemotherapeutics is increased and trapping of drug molecules can be achieved with high potency. Recent study has showed incorporation of PEG onto liposomes surface, could further enhance their in vivo stability, furthermore, PEG-stabilized liposomes exhibited a prolonged plasma half-life, as the surface-exposed PEG chains form a protective layer around liposomes to reduce clearance by macrophage in reticuloendothelial system [65]. In a mouse neuroblastoma model, Lee et al. demonstrated that liposome-based gold porphyrin nanoparticles composite could inhibit tumor growth with reduction of systematic toxicity induced by gold porphyrin [66, 67].

Dendrimers have also been demonstrated to be a good delivery system to carry the anti-cancer agent. Klutz et al. adopted dendrimer for gene delivery for treatment of neuroblastoma, and found they could effectively modulate immune response [68].

In terms of clinical application, liposomes are the most established systems used for drug delivery. Liposomal nanotherapeutics for cancer treatment have been on the market for more than a decade, whereas other liposomal drugs are in various stages of clinical development. Some examples of available drugs are: liposomal amphotericin B, liposomal doxorubicin, liposomal daunorubicin [69–71]. All the liposomal formulations have been shown to have higher efficacy and lower toxicity than non-liposomal preparations.

### Naked metallic nanoparticles

Among all the naked nanodrugs, silver nanoparticles (AgNPs) and iron oxide nanoparticles are the most widely studied. For centuries, silver has been utilized as an effective anti-microbial agent. With the advent of nanotechnology, silver can now be formulated into nanoparticles. They seem to have all the beneficial effects of silver compounds without associated toxicity. Indeed, many studies showed that AgNPs could be even more effective in bacterial killing through damaging bacterial cellular proteins and blocking the microbial respiratory chain system [1, 72–74]. Tian et al. revealed that topically applied AgNPs reduced both local as well as systemic inflammation in a burn wound model [75]. The anti-inflammatory action was also confirmed also in a peritoneal adhesion model in mice [76]. The combined anti-bacterial and anti-inflammatory actions contributed to significantly faster wound healing. Regarding clinical applications, AgNPs has already been using in a commercially available dressing for burn wounds—Acticoat^®^ (Smith & Nephew) [77–80]. Other products containing nanosilver include silver-impregnated catheters [81], and surgical mesh [82].

For the superparamagnetic iron oxide nanoparticles (SPIONs), the main biological function is to assist delivery of small-molecule drugs or biological agents to the target site and limiting its exposure to healthy tissue. As SPION are magnetic, they can also be used as a contrast agent in MRI for disease diagnosis and treatment monitoring. In fact, several SPION formulations have been approved for clinical use including dextran-coated iron oxide (Feridex^®^) for liver and spleen imaging, and ferumoxytol for iron replacement therapy [83, 84]. SPIONs are safe and stable nanodelivery system to carry genes, including small interfering RNA (SiRNA), nucleic acids and plasmid DNA [85–87], as well as chemotherapy and proteins [88–90].

### Nanoparticles for vaccine/gene delivery

Current biological vaccines consist of polynucleotide vaccines, DNA vaccines, and plasmid vaccines. Some issues including efficient delivery of the vaccines molecules to target cell population, its localization to the nucleus of these cells, and ensuring that the integrity of the polynucleotides is maintained during delivery to the target site, are essential to maximize the biological efficacy [91]. In this regard, nanotechnology can serve as an efficient sustained release delivery system for loaded vaccines. The nanoparticles could release these vaccines molecules at a sustained rate leading to continuous gene expression. This preserves the level of vaccine molecules in blood and maintains continuous production of specific antibody.

### Nano-surgery

Advances in nanotechnology in recent years have resulted in a new concept termed nanoscale laser surgery. Cellular structures could be manipulated at nanoscale level using femtosecond (fs: one millionth of a billionth of a second) laser pulses. Femtolaser can selectively cut a single strand in a single cell. This means that even organelles inside a single cell could be removed without disrupting rest of cell. Thus, it may provide an ideal tool in ophthalmological surgery [92].

**Fig. 3:**
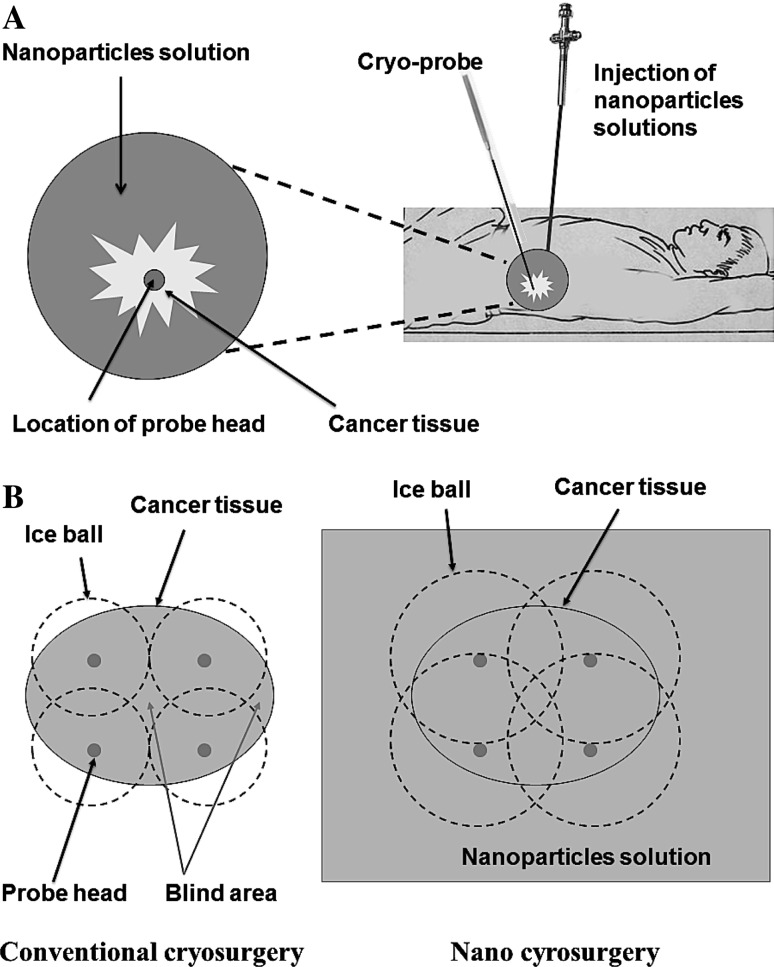
Schematic diagram showing the concept of nano-cryosurgery. **a** Specific nanoparticle solution is injected around cancer tissue to improve thermal conductivity, followed by insertion of cryo-probe under image guidance. **b** Super-conductive nanoparticles lower the freezing temperature and the freezing zone (*dashed line*) around cancer tissue when compared to conventional cryosurgery

On the other hand, nano-cryosurgery provides a new variation of an old concept. The principle of it lies in the loading of specific nanoparticles within and around cancer tissue. Their excellent cooling conductivity will contribute to the fast freezing at a lower temperature (Fig. 3). Cancer cell apoptosis induced by freezing action and curative efficacy was improved greatly [93]. Although the obstacles for nano-cryosurgery technology to be used in the clinic are to be overcome, it is believed to be a promising choice for tumor control in the future.

## Nanomedicine meets regenerative medicine

This subject engages in the study of application of nanomaterials or devices on damaged tissues or organs, aiming to promote tissue regeneration and repair with minimal scar and maximal function after injury. This field encompasses a vast area but we shall focus mainly on the aspects of skin and bone regeneration, as damages to these organs are the most commonly encountered.

**Fig. 4:**
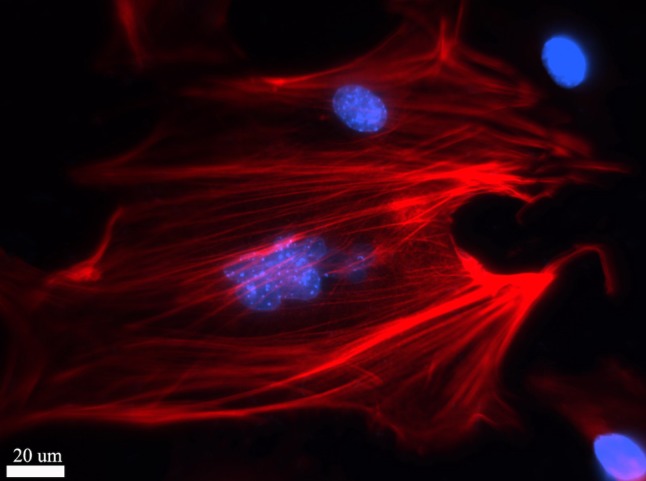
Immunocytochemistry staining of myofibroblast differentiated from mice embryonic fibroblasts under the influence of silver nanoparticles. *Red* α-SMA protein, *Blue* cell nuclear (DAPI staining)

Regarding to the regeneration and repair of skin and bone tissue using nanomaterials, intensive research work have been carried out trying to accelerate wound healing and recovery of mechanical function. In this regard, Liu et al. explored the use of AgNPs on skin excisional wound healing. The in vitro and in vivo experiments revealed that AgNPs could promote keratinocyte proliferation in the re-epithelization process, while they could drive the differentiation of fibroblasts into myo-fibroblast for wound contraction (Fig. 4) [94]. Further study demonstrated that the mechanical function in healed skin after treatment with AgNPs had similar elastic force, collagen deposition, as well as collagen fibrils alignment to normal skin [95]. These indicated that AgNPs could regulate remodeling process during skin tissue regeneration.

For bone repair, nanotechnology has contributed towards the design and construction of scaffolds using various materials, such as collagen, calcium sulfate, chitosan hydroxyapatite [96–99]. These can be further modified with the addition of nanoparticles like boron, growth factors, and/or stem cells [100–103]. The various nano-based scaffolds acted on bone matrix to promote recruitment of circulating stem cells, induce proliferation and eventual differentiation into mature osteoblasts.

Other applications of nanotechnology in regenerative medicine include repair of nerve injuries. Liu et al. investigated the feasibility of incorporating neurotrophin-3 and chondroitinase ABC onto electrospun collagen nanofibers for the treatment of spinal cord injuries. Their results showed accelerated nerve regeneration through provision of topographical signals and multiple biochemical cues arising from both nanofibrous scaffolds and cytokines [104].

Another approach was demonstrated by Ellis-Behnke et al. He used self-assembling peptides to explore their roles in axonal regeneration after injury in the central nervous system. In an optic tract injury model, injection of self-assembling peptide locally resulted in regeneration of axons through the site of acute injury, with subsequent functional return of vision [105].

The use of self-assembling nanofibrous scaffolds was also found to be effective in modulation of stem cells in wounds. Segers et al. showed that addition of stromal cell-derived factor-1 (SDF-1) in self-assembling nanofibrous scaffolds promoted stem cell recruitment and improved cardiac function in myocardial infarction model [106].

## Toxicology: should we be concerned?

With the wide use of various nanomaterials in the biomedical field, the issue of potential toxicity is of concern. Compared with conventional materials, nanoparticles can gain easy access to cells, tissue, even organs. Many researchers have been already engaging in cytotoxicity evaluation in various cells, to investigate and estimate the potential toxicity induced. Most of these studies are in vitro toxicity studies, and the general consensus is that nanomaterials at low dose will not cause significant cytotoxicity. However, just like most other agents or drugs, increasing concentration or exposure time of nanomaterials will result in observable cytotoxicity. Furthermore, the toxicity thresholds for various cell types are also different.

In addition to in vitro toxicity studies, in vivo toxicity studies need also be carried out. Oral and intravenous injections are the main administration routes of nanomaterials. Compared to in vitro toxicity studies, there are significantly fewer reports on in vivo toxicity of nanomaterials. Relevant clinical toxicity reports are even more sparse. Furthermore, between the biomedical efficacy and potential toxicity, a balance point probably exists, which can surely be swung towards the safety side by technological advances. For example, through precise modulation of size and shape of nanomaterials, the toxic effect can be modified. Furthermore, with the development of tissue engineering and scaffolds as well as nanodelivery system, the sustained release of nanomaterials in specifically targeted organs in the body can be achieved, thus reducing systemic toxicity effect. Nonetheless, it is imperative that for a new nanoproduct to be introduced, vigorous testings need to be conducted to ensure safety to our patients.

## Conclusion

Nanomedicine is now fully into our daily life and has brought innovation to current diagnostic and therapeutic approaches in clinical medicine. In the next decade, newer materials, technologies and methods will be emerging to promote further development of nanomedicine. Meanwhile, more research work in this field will make this subject more mature and eventually serve as a more effective tool for our healthcare system.
